# Transcriptional responses of invasive and indigenous whiteflies to different host plants reveal their disparate capacity of adaptation

**DOI:** 10.1038/srep10774

**Published:** 2015-06-04

**Authors:** Hong-Xing Xu, Yue Hong, Min-Zhu Zhang, Yong-Liang Wang, Shu-Sheng Liu, Xiao-Wei Wang

**Affiliations:** 1 Ministry of Agriculture Key Laboratory of Agricultural Entomology, Institute of Insect Sciences, Zhejiang University, Hangzhou 310058, China

## Abstract

The whitefly *Bemisia tabaci* contains more than 35 cryptic species. The higher adaptability of Middle East-Asia Minor 1 (MEAM1) cryptic species has been recognized as one important factor for its invasion and displacement of other indigenous species worldwide. Here we compared the performance of the invasive MEAM1 and the indigenous Asia II 3 whitefly species following host plant transfer from a suitable host (cotton) to an unsuitable host (tobacco) and analyzed their transcriptional responses. After transfer to tobacco for 24 h, MEAM1 performed much better than Asia II 3. Transcriptional analysis showed that the patterns of gene regulation were very different with most of the genes up-regulated in MEAM1 but down-regulated in Asia II 3. Whereas carbohydrate and energy metabolisms were repressed in Asia II 3, the gene expression and protein metabolisms were activated in MEAM1. Compared to the constitutive high expression of detoxification genes in MEAM1, most of the detoxification genes were down-regulated in Asia II 3. Enzymatic activities of P450, GST and esterase further verified that the detoxification of MEAM1 was much higher than that of Asia II 3. These results reveal obvious differences in responses of MEAM1 and Asia II 3 to host transfer.

Plants and insect herbivores are perpetually at battle in the evolutionary army race. Plants evolve various ways to become less hospitable to insect herbivores, while insect herbivores develop sophisticated defense systems to cope with their host plants or to find novel ways to exploit new hosts^1^. During the evolution, plants have evolved morphological barriers, including plant trichomes, surface waxes, and tissue thickness, to inhibit insect settlement on the plant^2^. In addition, plants’ primary and second metabolites, such as allelochemicals, nonprotein amino acids, and plants’ varying levels of nutrients (host quality), may reduce insect survival, growth and fecundity^3^,^4^,^5^. In parallel, insects also develop multiple behavioral and physiological mechanisms to overcome plant defenses, allowing them to survive on certain species of plants or even establish compatible interactions with multiple plant species^6^,^7^,^8^.

The whitefly *Bemisia tabaci* (Gennadius) (Hemiptera: Aleyrodidae) is a species complex with global distribution and extensive genetic diversity^9^,^10^,^11^. In this species complex, the Middle East-Asia Minor 1 (MEAM1, previously referred as the ‘B biotype’) species has been spreading and displacing the native whitefly species in many tropical and subtropical regions in the last 20 years. It causes excessive crop losses through directly feeding and transmission of plant viruses^12^,^13^,^14^. Previously, asymmetric mating interactions with indigenous whiteflies and beneficial interactions with plant viruses have been shown to be crucial in the invasion of MEAM1^15^,^16^. In addition, MEAM1 has a wider host range and higher host adaptability than many indigenous species of the *B. tabaci* complex, which may play an important role in the process of whitefly invasion as well^12^,^14^,^17^,^18^.

To date, many experimental studies have compared the performance of MEAM1 and other whitefly species on different plant species. Zang *et al.*^17^ showed that on tobacco MEAM1 whiteflies can develop successfully from egg to adult, whereas no individuals of Asia II 3 can. Xu *et al.*^18^ showed that MEAM1 whiteflies performed better than the indigenous Asia II 1 whiteflies on several commonly cultivated crop species. In addition, MEAM1 whiteflies have the ability to gradually acclimatize to unsuitable host plants through a series of intermediate hosts^19^. Such plasticity in host utilization may help MEAM1 to increase its population in areas where only poor or unsuitable host plants are available, and thus aid in its invasion. Thus, it is of interest to examine how MEAM1 and indigenous whiteflies respond after being transferred from a favorite host to an unsuitable host plant.

The performance of phloem-feeding insects is closely related to the amino acid and carbohydrate content of phloem sap of their host plants^20^,^21^. A Florida strain of sweet potato whitefly was found to have an expanded host range probably due to the fact that it processed more phloem sap so as to have access to more amino acids which allowed it to expand its host range^22^. As the nutritional quality of the phloem sap changed in water stressed plant, especially when the carbohydrate concentrations were higher, feeding rates of whitefly on water stressed plants significantly declined^23^. The feeding behaviors of piercing-sucking insect varied on different plants, which could be partly explained by the change of amino acid concentrations among the phloem sap of various plants^24^,^25^,^26^. Furthermore, MEAM1 performed better on virus infected tobacco plants than on uninfected plants, because whiteflies were able to achieve a more balanced nutritional assimilation from virus-infected plants^27^. As the nutritional quality of phloem sap varies in different plants, it’s interesting to investigate the mechanisms for *B. tabaci* to adapt to different host plant diets.

Insect resistance to plant chemicals is another key determinant for the adaptability of herbivorous insects. Overproduction of detoxification enzymes in insects is a main mechanism of metabolic resistance to plant toxins^6^. In general, cytochrome P450 monooxygenases (P450s), carboxylesterases (COEs) and glutathione S-transferases (GSTs) are widely employed by insects as the main detoxification mechanisms to allelochemicals^6^,^28^,^29^. Constitutive and inducible defences are two defence strategies utilized by different whitefly species. MEAM1 utilizes inducible defense in response to high levels of glucosinolates as some detoxification genes are significantly induced during exposure to indolic glucosinolates, while Mediterranean (MED, previously referred to as the ‘Q biotype’) species utilizes constitutive defence as most of the detoxification genes are constitutively expressed at a higher level in MED than in MEAM1 species^30^. However, an overall picture of how *B. tabaci* adapts to different host plants is still lacking.

The recently available whitefly transcriptome sequences^31^,^32^,^33^ have provided us unprecedented opportunities to investigate the global transcriptional response of whiteflies to different host plants. In this study, we compared the transcriptional responses of MEAM1, one of the most important invasive pest in China^16^, and Asia II 3 which is a native whitefly species and had almost been replaced by MEAM1 in China^17^, to host plant transfer (from cotton to tobacco). It has been known that cotton is a suitable host for both MEAM1 and Asia II 3; and these two species have similar performance on cotton. However, while MEAM1 can survive poorly on tobacco, all Asia II 3 whiteflies end up dead after several days on tobacco. Therefore, it is an adaptation process for MEAM1 and a non-host process for Asia II 3, when they were transferred from cotton to tobacco. Through this comparison, we hope to reveal the adaptation or tolerance mechanism of the invasive MEAM1 to an unsuitable host plant. First, we compared the performance of MEAM1 and Asia II 3 whiteflies transferred from cotton to tobacco. Second, we analyzed the gene expression variations of MEAM1 and Asia II 3 in response to host plant transfer using the Illumina sequencing technology. Last, we measured the detoxification enzyme activities of MEAM1 and Asia II 3 transferred from cotton to tobacco separately to illustrate the function of different detoxification systems during host plant transfer.

## Results

### Mortality and fecundity of whiteflies following host plant transfer

**Figure 1:**
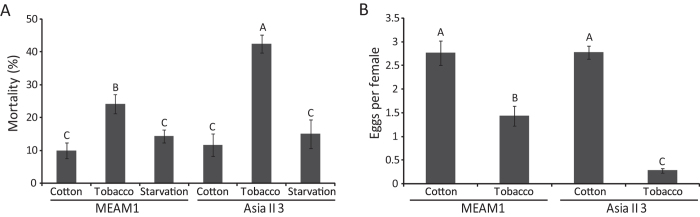
The performance of whiteflies following transfer to cotton, tobacco and empty clip cage. Whiteflies were transferred from cotton to cotton, tobacco or empty clip cage for 24 h. (**A**) The percentage of mortality. (**B**) The average number of eggs per female. Data shown are mean ± SE, n = 12. Different letters above the bars indicate significant differences between treatments at *p* < 0.05.

The mortality and fecundity of MEAM1 and Asia II 3 whiteflies were determined when they were transferred from cotton to tobacco and from cotton to cotton (as a control), respectively. After they were transferred to cotton plants for 24 h, the mortalities of MEAM1 and Asia II 3 whiteflies were comparable (about 10%), suggesting that cotton is equally suitable to both species. However, after transfer to tobacco for 24 h, the mortality of Asia II 3 (40%) was significantly higher than that of MEAM1 (24%) (Fig. 1A). To examine whether the higher mortality of whiteflies on tobacco was due to starvation (the insects may not feed on tobacco), whiteflies were also transferred into empty clip cages. Interestingly, the mortalities of both Asia II 3 and MEAM1 whiteflies in empty clip cage for 24 h were comparable to their mortalities on cotton (Fig. 1A), suggesting that the high mortality of whiteflies on tobacco was not due to starvation but due to feeding/staying on tobacco. Likewise, while the mean number of eggs per female on tobacco laid by each of the two species was significantly lower than that on cotton, the fecundity of MEAM1 on tobacco was five-fold higher than that of Asia II 3 (Fig. 1B). These data indicate that: i) tobacco is a less suitable plant for both MEAM1 and Asia II 3 whiteflies; and ii) compared with MEAM1, Asia II 3 performed poorer after being transferred from cotton to tobacco.

### Gene expression of MEAM1 and Asia II 3 whiteflies following host plant transfer

To reveal how the two whitefly species respond to host plant transfer, four samples: MEAM1 on cotton (MC), MEAM1 on tobacco (MT), Asia II 3 on cotton (AC) and Asia II 3 on tobacco (AT) were collected and sequenced, respectively. Approximately 6.0 million raw tags were obtained for each library and up to 39.05% (22,547) of the sequences in the reference database could be unambiguously identified by unique tags (Table 1). To identify the differentially expressed genes (DEGs) after transfer to tobacco, two comparisons were performed: 1) MT vs. MC; and 2) AT vs. AC.

**Figure 2:**
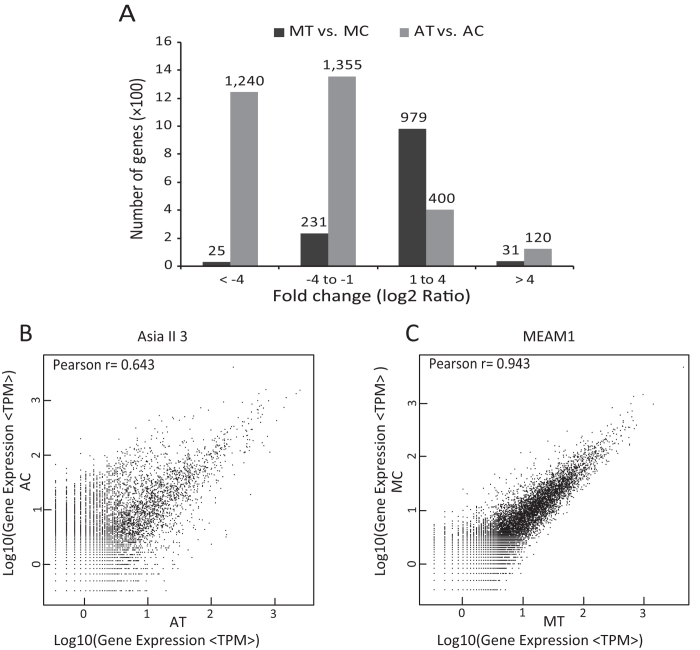
Changes in gene expression profiles of MEAM1 and Asia II 3 on cotton and tobacco. (**A**) The number and fold change (log_2_ Ratio) distribution of differentially expressed genes. Correlation analysis of AT vs. AC (**B**) and MT vs. MC (**C**). The Pearson correlation coefficient for each comparison is shown in the upper left corner of the plot.

The gene regulation patterns of MEAM1 and Asia II 3 whiteflies were very different after they were transferred to tobacco for 24 h. Among the 57,741 genes in the transcriptome of MEAM1, 1,266 genes were found differentially expressed (False discovery rate, FDR < 0.001 and absolute value of log_2_^Ratio^ ≥ 1), with 79.8% (1,010) of genes up-regulated (Fig. 2A; Table S1). However, among the 52,535 genes in the transcriptome of Asia II 3, more genes (3,115) were differentially expressed and 83.3% (2,595) of DEGs were down-regulated after feeding on tobacco for 24 h (Fig. 2A; Table S2). Moreover, while 95.6% (1,210) genes were regulated by one- to four-fold in MEAM1, about half of the DEGs of Asia II 3 were regulated by four- to twelve-fold (Fig. 2A). The Pearson correlation coefficients of the two libraries were 0.643 for AT vs. AC and 0.943 for MT vs. MC, suggesting that host plant transfer had a much larger effect on the gene expression profile of Asia II 3 than that of MEAM1 (Fig. 2B,C).

To validate the DGE data, quantitative reverse transcription PCR (qRT-PCR) was used to examine the expression profile of 50 randomly selected DEGs in MT vs. MC and AT vs. AC. Out of the 50 genes selected, 45 showed a concordant direction of change for both DGE and qRT-PCR (Table S3). Discrepancies in the data obtained from DGE and qRT-PCR have been described previously^34^,^35^, nevertheless qRT-PCR confirmed the up- and down-regulation of genes detected by DGE analysis and proved the validation of DGE analysis.

### GO and KEGG pathway analysis

**Figure 3:**
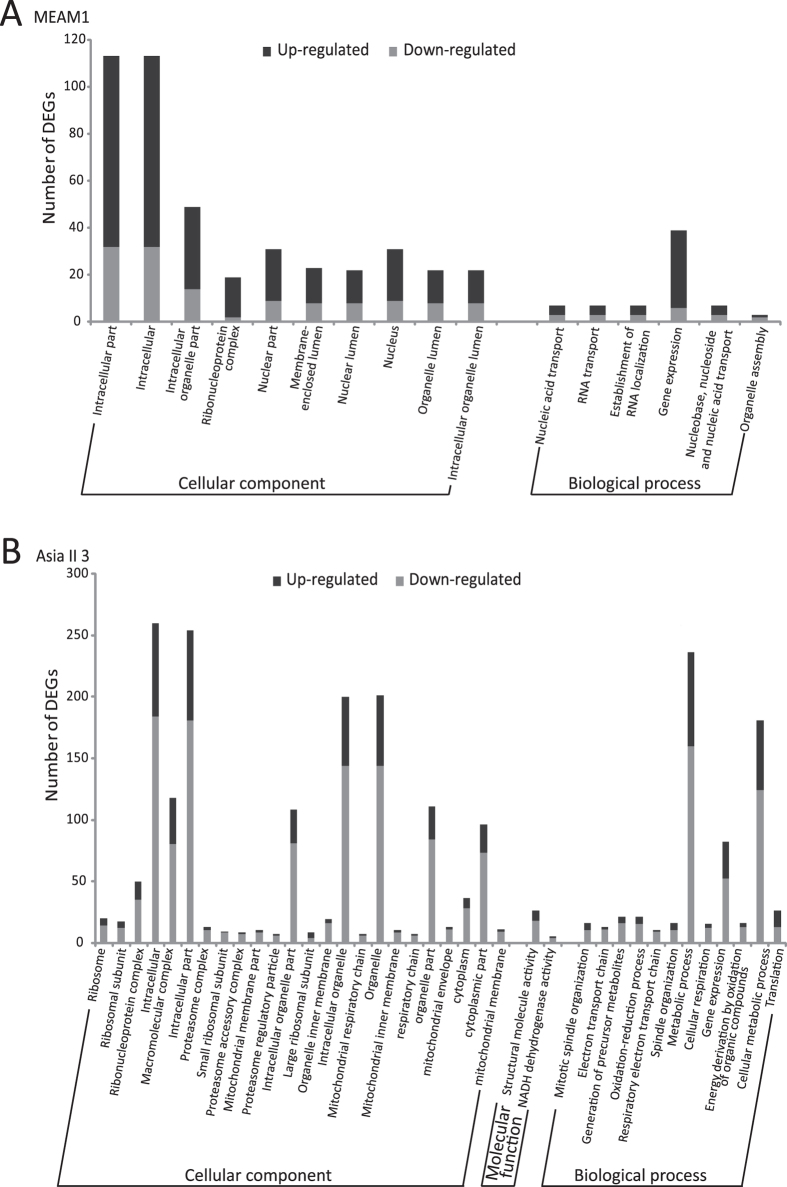
Gene ontology terms enriched with DEGs after host transfer. (**A**) GO terms enriched with DEGs in MEAM1. (**B**) GO terms enriched with DEGs in Asia II 3. The functions of genes identified cover three main categories: cellular component, molecular function and biological process.

The Gene Ontology (GO) analysis was used to classify the functions of all DEGs in MEAM1 and Asia II 3 whiteflies feeding on different host plants. Among the 1,266 DEGs in MEAM1 species, 126 genes were mapped to the category of Cellular component, 156 genes to Molecular function, and 140 genes to Biological process. Among the 3,115 DEGs in Asia II 3 species, 306 genes were mapped to the category of Cellular component, 356 genes to Molecular function, and 326 genes to Biological process. GO enrichment analysis showed that in MEAM1, 10 GO terms were enriched with DEGs in the category of Cellular component and 6 GO terms were enriched with DEGs in Biological process category; however, there were no terms enriched in the Molecular function category (Fig. 3A). Meanwhile a total of 38 GO terms were enriched with DEGs in Asia II 3 species with ‘intracellular’, ‘structural molecule activity’ and ‘metabolic process’, having most DEGs in the three main categories respectively (Fig. 3B).

The Kyoto Encyclopaedia of Genes and Genomes (KEGG) pathway analysis showed that 225 DEGs were mapped to 138 pathways in MT vs. MC comparison group, and 469 genes in AT vs. AC group were mapped to 186 pathways. Pathway enrichment analysis showed that 8 pathways in MEAM1 and 24 pathways in Asia II 3 were enriched with DEGs after host plant transfer (Table 2).

### Feeding on tobacco repressed the carbohydrate and energy metabolism of Asia II 3

**Figure 4:**
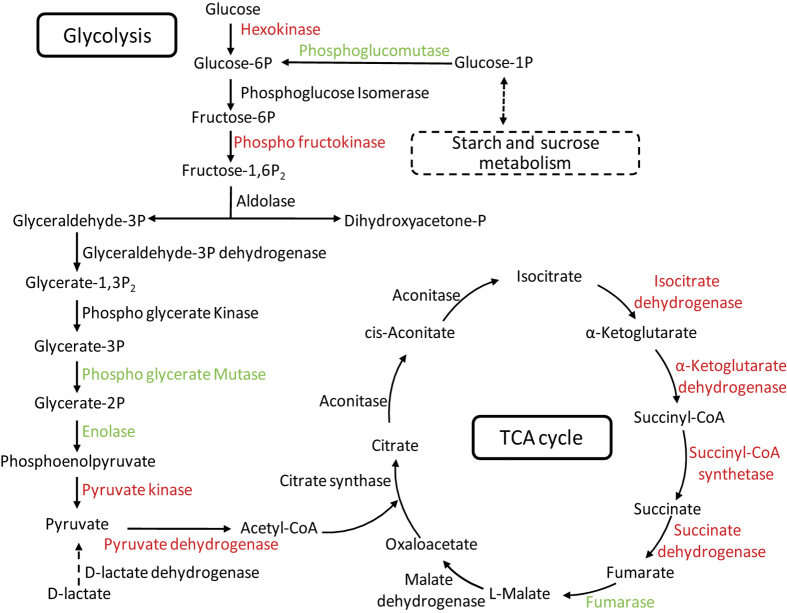
Transcriptional changes of genes in the glycolysis and TCA pathways in Asia II 3. The DEGs encoding enzymes involved in glycolysis and TCA cycle are shown. Up-regulated genes are highlighted in green while the down-regulated genes are highlighted in red.

In Asia II 3, DEGs were significantly enriched in four carbohydrate metabolism pathways and one energy metabolism pathway including ‘Oxidative phosphorylation (OXPHOS)’, ‘Citrate cycle (TCA cycle)’, ‘Glycolysis’, ‘Propanoate metabolism’ and ‘Pentose phosphate pathway’ (Table 2, Table S4). In our data, the expression levels of hexokinase, phosphofructokinase and pyruvate kinase genes, which play key roles in glycolysis pathway^36^, were all significantly down-regulated in AT vs. AC, indicating that glycolysis was strongly inhibited in Asia II 3 after feeding on tobacco for 24 h (Fig. 4, Table S4). It is noteworthy that phosphoglucomutase (PGM) was up-regulated in Asia II 3 whiteflies, suggesting that whiteflies can adjust their carbohydrate metabolism to adapt to the different plant nutritional composition. In addition, the increase of transcription of phosphoglyceratemutase and enolase, which catalyze the synthesis of phosphoenolpyruvic acid (PEP) and release -OH, was also observed in glycolysis pathway in AT vs. AC.

In eukaryotes, the TCA cycle and OXPHOS is a vital part of energy metabolism to supply ATP for organisms^37^. TCA cycle was repressed in AT vs. AC as 4 genes were significantly down-regulated including isocitrate dehydrogenase, 2-oxoglutarate dehydrogenase E1 component, succinyl-CoA synthetase and succinate dehydrogenase (Fig. 4, Table S4). Moreover, because of the down-regulation of pyruvate kinase and pyruvate dehydrogenase genes in AT vs. AC (Fig. 4, Table S4), the TCA cycle would be affected due to the lack of raw material pyruvate^38^ in Asia II 3 when they fed on tobacco for 24 h. The mitochondrial electron transport chain was also disturbed seriously and the synthesis of ATP was inhibited in Asia II 3 as most of DEGs (35 out of 38) mapped to OXPHOS pathway were down-regulated in AT vs. AC including NADH dehydrogenase, succinate dehydrogenase, cytochrome c reductase and cytochrome c oxidase (Table S4). Collectively, these data indicate that the carbohydrate and energy metabolism are significantly repressed in Asia II 3, which may lead to the death of Asia II 3 whiteflies on tobacco.

### Gene expression and protein metabolism in MEAM1 whiteflies after host plant transfer

In MEAM1, the results of GO enrichment analysis showed that six biological processes related to RNA metabolism and gene expression were enriched with DEGs (Fig. 3A). Among the 39 DEGs in ‘gene expression’ GO term, 33 DEGs were up-regulated, suggesting that gene transcription might be activated in MEAM1 after host plant transfer. In contrast, 52 DEGs (63%) in the ‘gene expression’ GO term in Asia II 3 were significantly down-regulated (Fig. 3B). The difference between MEAM1 and Asia II 3 in ‘gene expression’ can also be correlated to the mechanism that more genes were up-regulated in MEAM1.

Ribosomes are basic for RNA translation and cellular viability, so the transcription levels of ribosomal genes were used to estimate the cell injury provoked by various stresses^39^,^40^. Upon feeding on tobacco, the expressions of 28 ribosome genes of Asia II 3 were significantly changed, of which 64% (18) were down-regulated and 36% (10) were up-regulated (Table 2, Table S5). In contrast, ‘Ribosome’ pathway was not significantly regulated in MEAM1 and only 3 DEGs were mapped to this pathway with 2 genes up-regulated. These results suggest that the protein synthesis process is not significantly affected in MEAM1 but is seriously repressed in Asia II 3, which is consistent with the stronger tolerance of MEAM1 to tobacco. A recent study in *Chironomus riparius* showed that the ribosomal RNA transcription was significantly down-regulated by the high concentration of butyl benzyl phthalate (BBP)^40^. Therefore, the inhibition of ribosomal gene expression in Asia II 3 may be caused by taking some plant allelochemicals in tobacco phloem sap.

Proteasome and lysosome are two different but functionally coupled protein degradation systems^41^,^42^. The main function of the proteasome is to degrade intracellular unneeded or damaged proteins by ubiquitin-dependent protein degradation^43^. The peptides generated from lysosomal degradation and ubiquitin-proteasome system will be further degraded into shorter amino acid sequences and used in synthesizing new proteins^44^,^45^,^46^. KEGG pathway analysis showed that the DEGs were enriched in ‘proteasome’ pathway for both MEAM1 and Asia II 3 (Table 2), suggesting that the protein degradation metabolism was regulated in both whitefly species when they were transferred onto tobacco for 24 h. Interestingly, ‘proteasome’ pathway was activated in tobacco-feeding MEAM1 whiteflies as indicated by 4 up-regulated genes in this pathway, but repressed in Asia II 3 as 11/14 DEGs in ‘proteasome’ pathway were down-regulated (Table S6). Moreover, most of the DEGs in the ‘lysosome’ and ‘protein digestion and absorption’ pathway of MEAM1 and Asia II 3 were down-regulated, indicating that the ingested tobacco phloem sap proteins cannot be efficiently utilized when whiteflies were transferred to feed on tobacco.

### Differential regulation of detoxification genes in Asia II 3 and MEAM1

**Figure 5:**
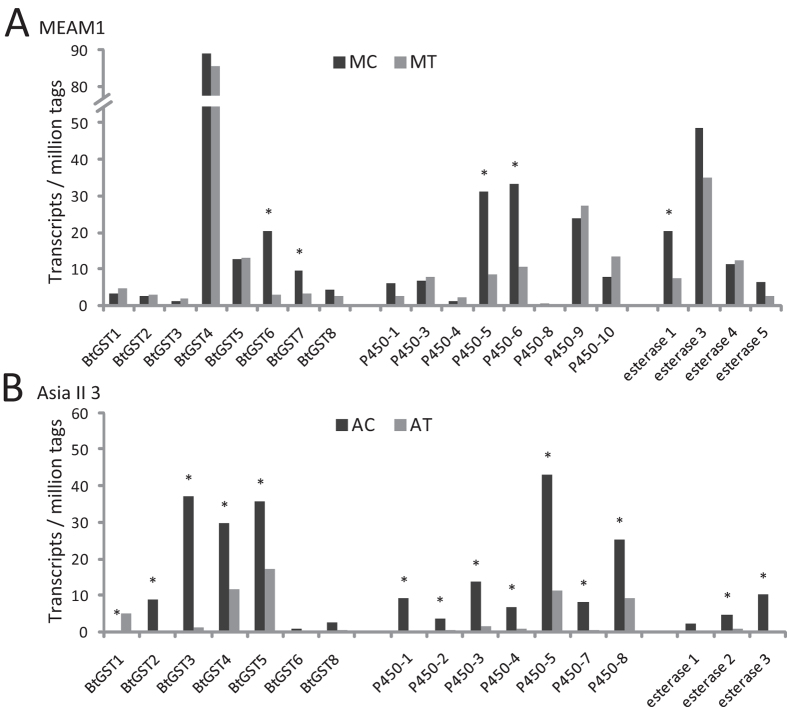
Transcription profiles of 23 detoxification genes from the GST, P450 and esterase families. MEAM1 (**A**) and Asia II 3 (**B**) whitefly adults were transferred from cotton to feed on tobacco for 24 h. ‘*’ denotes the differently expressed genes judged by FDR < 0.001 and the absolute value of log_2_^Ratio^ ≥ 1.

Detoxification enzymes of insects, typically including P450s, GSTs and COEs, play important roles in insecticide resistance, tolerance to plant toxic compounds and metabolism of endogenous compounds^6^,^28^,^47^. Therefore, we screened all homologous genes of P450s, GSTs and COEs in our sequencing data and a total of 23 genes with putative function of detoxification were found (Table S7). Among the 23 detoxification genes in MEAM1 and Asia II 3, different patterns of gene expression were found. While most of the detoxification genes in MEAM1 were not substantially changed when they fed on tobacco for 24 h, the majority of detoxification genes in Asia II 3 were drastically down-regulated to an extremely low level (Fig. 5). When MEAM1 whiteflies were transferred to feed on tobacco for 24 h, only 5 detoxification genes (*BtGST6*, *BtGST7*, *P450-5*, *P450-6* and *esterase1*) were differentially expressed (Fig. 5A, Table S7). However, in Asia II 3, 13/17 detoxification genes were significantly down-regulated after feeding on tobacco for 24 h (Fig. 5B, Table S7). In addition, KEGG pathway enrichment analysis showed that 15 DEGs were enriched in ‘Metabolism of xenobiotics by cytochrome P450’ and ‘Drug metabolism - cytochrome P450’ pathways in AT vs. AC, suggesting that detoxification metabolism of Asia II 3 was significantly suppressed after transferring on tobacco (Table 2).

Among the 20 detoxification genes analyzed in MEAM1, *BtGST4*, *BtGST5*, *P450-9*, *esterase3* and *esterase4* were consistently expressed at a high level (10 < TPM < 90) and the changes following host transfer were not significant (Fig. 5A, Table S7). However, in Asia II 3, seven of these detoxification genes (*BtGST2*, *P450-1*, *P450-2*, *P450-4*, *P450-7*, *esterase2* and *esterase3*) were down-regulated to an extremely low level (TPM < 1) following host transfer (Fig. 5B, Table S7), which was much lower than the expression level of their homologous genes in MEAM1. These data indicate that the detoxification system of MEAM1 is less affected by host plant transfer. Although no significantly up-regulated detoxification gene was founded in MEAM1 species after host plant transfer, the consistently high expression of detoxification genes may be an important reason why MEAM1 can feed on a wide range of host plants.

### Activities of the detoxification enzymes in MEAM1 and Asia II 3 whiteflies

**Figure 6:**
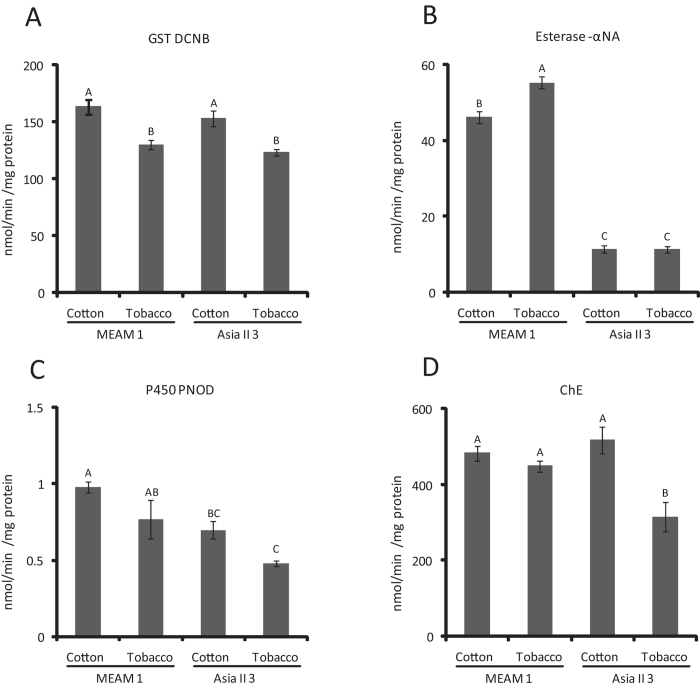
Activities of major detoxification enzymes of MEAM1 and Asia II 3 whiteflies on cotton and tobacco. (**A**) GST activity towards DCNB. (**B**) Esterase activity towards a-NA. (**C**) P450 activity towards PNOD. (**D**) ChE activity. Each assay was repeated three times. The results are shown as mean ± SE, and the different letters above the bars indicate significant differences (*p* < 0.05).

To further reveal the differences between MEAM1 and Asia II 3 whiteflies, the enzymatic activities of three major detoxification enzymes, cytochrome P450 monooxygenases, GSTs and esterases were monitored when the MEAM1 and Asia II 3 whiteflies were transferred onto tobacco for 24 h. The GST activities of both MEAM1 and Asia II 3 whiteflies were decreased by about 20% when they were transferred from cotton to tobacco (Fig. 6A). For MEAM1, the activity of esterase towards α-Naphthol (α-NA) was elevated significantly when they were transferred to tobacco, whereas the esterase activity of Asia II 3 was much lower than that of MEAM1 on cotton and was not significantly changed after feeding on tobacco for 24 h (Fig. 6B). For cytochrome P450 monooxygenases activity, the p-nitroanisole O-demethylase (PNOD) activity of both MEAM1 and Asia II 3 were decreased by about 25% when they were transferred to tobacco. However, the PNOD activity of MEAM1 was about 1.4 times higher than that of Asia II 3 on cotton and 1.6 times higher on tobacco (Fig. 6C). Although choline esterase (ChE) was not considered as a detoxification enzyme, the activity of ChE has an important role in nicotine poisoning. Figure. 6D showed that host plant transfer did not affect the ChE activity of MEAM1 but inhibited the ChE activity of Asia II 3 significantly. Taken together, these data demonstrate that transferring to tobacco has significant effects on both the invasive and indigenous whiteflies and the detoxification capability of MEAM1 is much higher than that of Asia II 3.

## Discussion

The invasive whitefly species are often known to have a wider host range than indigenous species, and such differences in host range between invasive and indigenous species are expected to aid in biological invasions^12^,^14^. Our work demonstrates striking differences in response to host shift between the two whitefly species, including insect performance, gene transcription and enzymatic activities. First, the performance of MEAM1 transferred from cotton to tobacco for 24 h was better than that of Asia II 3. Second, the gene expression profiling showed that many more DEGs with larger fold-change were found in Asia II 3 than in MEAM1. Third, the KEGG pathway enrichment analysis showed that carbohydrate and energy metabolism pathways were repressed in Asia II 3 but not affected in MEAM1. Fourth, gene expression and protein metabolism were induced in MEAM1 whiteflies as the gene expression and proteasome pathway were activated. Finally, the activities of most detoxification enzymes were changed slightly in MEAM1 but were inhibited considerably in Asia II 3 after feeding on tobacco for 24 h. These results further indicate that the invasive MEAM1 species has stronger adaptability than the indigenous Asia II 3 species on unsuitable host plant during invasion and spread.

In our study, both MEAM1 and Asia II 3 whiteflies were adversely affected when they were transferred from cotton to tobacco. The increase of whitefly mortality on tobacco could be caused by the following factors: i) starvation as a result of no feeding, ii) adverse effects resulted from tobacco allelochemicals. The physical barrier of leaves such as leaf trichome and trichome exudates hinder insects from searching suitable feeding site smoothly^48^, so the different physical conditions of cotton and tobacco leaves are important reasons for the higher mortality of whiteflies on tobacco. However, even though the physical barrier of leaves may affect whitefly’s movement, observations with EPG showed that both MEAM1 and Asia II 3 whiteflies could feed on tobacco successfully^49^. In addition, when MEAM1 or Asia II 3 whiteflies were kept in empty clip cages without feeding for 24 h, the mortality was much lower than that on tobacco (Fig. 1). It is therefore assumed that the increased mortality of whiteflies on tobacco is most likely caused by the adverse effects of having access to tobacco allelochemicals.

In response to plant allelochemicals, MEAM1 whiteflies induced adaptive or defence mechanism, which was considered as a ‘optimal defence strategy’ for their survival^30^. In our study, most of the DEGs in MEAM1 were up-regulated, which implied that MEAM1’s adaptive or defence mechanism were induced when they were transferred to tobacco. However, similar mechanisms maybe not worked or existed in Asia II 3 because most of the DEGs were significantly down-regulated in Asia II 3 whiteflies. In general, our findings support the argument that the invasive MEAM1 whiteflies have stronger adaptability than Asia II 3 whiteflies. When encountering unsuitable host plants, the MEAM1 whiteflies can activate adaptive or inducible defence mechanism, which may contribute to their gradual adaption to unfavorable hosts. As our results only analyzed how MEAM1 and Asia II 3 whiteflies respond to host plant transfer in the initial short period of time (24 h), further studies are needed to analyze the long-term adaptive mechanisms of MEAM1 feeding on tobacco.

In this study, we stressed the whiteflies by transferring them from cotton to tobacco and analyzed the molecular responses of different whitefly species to the new host plant. Although the pathways related to digestive system were not significantly changed, the most basic and important carbohydrate and energy metabolism pathways in every organism, such as ‘Glycolysis’, ‘pyruvate metabolism’, ‘TCA cycle’ and ‘Oxidative phosphorylation’ pathways, were all repressed significantly in Asia II 3 but not affected in MEAM1. Therefore, genes involved in nutrition supply in MEAM1 are more tolerant to host changes and the inhibition of carbohydrate and energy metabolism in Asia II 3 may be another important reason for the poor performance of Asia II 3 on tobacco.

Insects have evolved various strategies for dealing with plant allelochemicals^29^. Usually, ingestion of plant toxins frequently causes insects to induce detoxification genes. For example, in *Helicoverpa armigera*, cytochrome P450 transcripts were induced by gossypol, deltamethrin and phenobarbital^50^,^51^. Moreover, specific GST genes in MED whitefly were significantly induced during exposure to high levels of indolic glucosinolate^30^. However, constitutive defense is also common in insects and plays an important role in the early stage of encounter with stress before the inducible defence is activated. In this study, except one GST gene, no other major detoxification genes (P450, GST and esterase) were significantly induced in either MEAM1 or Asia II 3 species when they were transferred to feed on tobacco for 24 h. Therefore, the constitutive defense might be the main defense mechanism for whiteflies in the first 24 h after host plant transfer. Furthermore, most of the detoxification genes in Asia II 3 whitefly were significantly down-regulated. As detoxification activity need energy, it seems that the down-regulation of detoxification genes in Asia II 3 is not just because of the direct effect of plant allelochemicals, but the attenuated energy supply in Asia II 3. As the plant allelochemicals are mixtures and vary in different plants, the inducible defense in response to plant allelochemicals may need longer time compared with the inducible defense to single component pesticide. Although no detoxification gene was induced, the constitutive expression of the detoxification genes was much higher in MEAM1 than in Asia II 3 (e.g. *BtGST4* and *esterase3*, Fig. 5), which may be important for the survival of MEAM1. The results of P450 and GST enzyme activity assays further support this inference. What’s more, the high esterase and ChE activity in MEAM1 may play an important role during its adaptation to unsuitable host plant.

## Conclusion

In this study, we analyzed the transcriptional responses of an invasive and an indigenous whitefly to different host plants using high-throughput sequencing. Our data showed that the gene regulation patterns of MEAM1 and Asia II 3 whiteflies were very different. Most of DEGs were up-regulated in MEAM1 whereas down-regulated in Asia II 3. Carbohydrate and energy metabolism was also repressed in Asia II 3 by interfering with the glycolysis, TCA cycle and oxidative phosphorylation pathway. However, genes involved in nutrition supply in MEAM1 were more tolerant to host changes and the activation of gene expression pathway facilitated the adaptation of MEAM1 to tobacco. Moreover, compared to the slight changes of detoxification genes in MEAM1, most of detoxification enzymes in Asia II 3 were drastically repressed. Altogether, this study provides the first overall picture underlying the wide host plant range of MEAM1 whiteflies.

## Methods

### Insect cultures and host plants

The stock cultures of the invasive MEAM1 (mitochondrial cytochrome oxidase 1 GenBank accession no. GQ332577) and indigenous Asia II 3 (mitochondrial cytochrome oxidase 1 GenBank accession no. DQ309076) whiteflies were separately maintained on cotton at 27 ± 1 °C, a photoperiod of 14 h light: 10 h darkness and 70 ± 10% relative humidity^16^,^52^. Two species of host plants, cotton (*Gossypium hirsutum* (Malvaceae) cv. Zhe-Mian 1793) and tobacco (*Nicotiana tabacum* cv. NC89) were cultivated singly in a greenhouse at 25 ± 3 °C, a photoperiod of 14 h light : 10 h darkness. All plants used in experiments were at four-to-six fully extended true-leaf stage and all experiments were conducted in climate chambers at 27 ± 1 °C, 14 h light : 10 h darkness and 70 ± 10% relative humidity.

### Host plant transfer assays

The sub-culture of each of the two whitefly cryptic species was initiated with 60 newly emerged adult whiteflies (30 females and 30 males) and was maintained on cotton for 3 generations before the adult whiteflies were taken for experiments. As newly emerged whiteflies are extremely vulnerable, whiteflies used in the experiments were all female adults taken 24 h after emergence. For each of the two whitefly species, three treatments were conducted including transfer from cotton to cotton, from cotton to tobacco, and from cotton to an empty clip cage^53^. For each treatment, twelve replicates were tested. And for each replicate in a given treatment, 10 females were collected from the sub-culture and released into a clip cage on the abaxial surface of the cauline leaves of a tobacco or a cotton plant, or to an empty clip cage (as a control of starvation). Twenty four hours after host transfer, all the dead individuals were collected and counted to calculate the mortality. Meanwhile all the eggs on the leaves were counted with a stereoscope and the number of eggs per female was determined. Statistical significance between the treatments was evaluated using one-way ANOVA at a 0.05 level followed by LSD tests. All data analyses were conducted using the software STATISTICA 6.1 (StatSoft, Inc., Tulsa, USA).

### RNA isolation, DGE library preparation and Illumina sequencing

Four treatments were conducted including MEAM1 from cotton to cotton (MC), MEAM1 from cotton to tobacco (MT), Asia II 3 from cotton to cotton (AC) and Asia II 3 from cotton to tobacco (AT). For each treatment, approximately 1,000 adult whiteflies (12–24 h after emergence) were transferred. After 24 h, all live whiteflies were collected for RNA isolation. Two replicates were conducted for each of the treatments and processed independently. One replicate was used for DGE library preparation and Illumina sequencing, and the other was used for the qRT-PCR analysis. Total RNA was isolated using SV total RNA isolation system (Promega, Madison, USA) according to the manufacturer’s instructions. mRNA purification and DGE library preparation were conducted as previously described^35^. All the DGE libraries were sent to Beijing Genome Institute (Shenzhen, China) for Illumina sequencing.

### Tag annotation and data normalization for gene expression level

Clean tags were extracted from raw tag sequences by removing adaptor sequence and low-quality sequences. All tag mapping, annotation, data normalization and gene expression analysis were performed according to the previously published procedures^35^. Tags were annotated by mapping tag sequences to the transcriptome reference database of MEAM1 and Asia II 3 (available upon request). For tag mapping, mismatch was not allowed. All tags mapped to multiple genes were filtered out and the remaining tags were designed as unambiguous tags. The number of unambiguous tags for each gene was calculated and then normalized to TPM (number of transcripts per million tags) for differential gene expression analysis^34^,^54^.

### Analysis of differentially expressed genes

To identify the DEGs between cotton-feeding and tobacco-feeding whiteflies (MT vs. MC and AT vs. AC), a rigorous algorithm was used based on a method described previously^55^. FDR was used to determine the threshold of *P* value in multiple tests and analysis. FDR < 0.001 and the absolute value of log_2_^Ratio^ ≥ 1 were used as the threshold to judge the significance of gene expression difference. Pearson correlation coefficient was calculated to statistically assess the variations of the two comparison groups, MT vs. MC and AT vs. AC.

### GO and KEGG pathway analysis

To further understand the molecular events behind the genes expression, all DEGs were subjected to GO functional annotation using Blast2GO and mapped to terms in KEGG pathway database using Blastall software. Enrichment analysis was used to identify the significantly regulated KEGG pathways and GO terms by hypergeometric test of R program. Significantly enriched pathways were filtered out with the threshold of *P* value ≤0.05.

### qRT-PCR analysis

In order to confirm the results of the DGE analyses, the expression of 50 selected genes were measured using qRT-PCR. One replicate of total RNAs of the four treatments were served as templates to synthesize cDNAs using the SYBR^®^PrimeScript™ RT-PCR Kit II (Takara). qRT-PCRs were done on the ABI PRISM 7500 Fast Real-Time PCR System (Applied Biosystems) with SYBR-Green detection. Each gene was analyzed in triplicate and the average threshold cycle (Ct) was calculated. The relative expression levels were calculated with the 2^−ΔΔCt^ method. A house keeping gene β-actin was used for transcript normalization^35^.

### Detoxification enzyme activity assay

To assess the effect of host plant transfer on detoxification enzyme activities of the two whitefly species, total proteins were extracted from whiteflies. For each treatment, approximately 1,000 adults were collected and homogenized in 1 mL precooling-homogenization buffer (0.1 M sodium phosphate, 1 mM EDTA, 1 mM DTT, 1 mM PMSF and 10% glycerol, pH 7.8). The homogenate was centrifuged at 4 °C, 10,000 × g for 20 min and the supernatant was filtered using silica wool. The filtrate was then centrifuged at 4 °C, 100,000 × g for 60 min and the supernatant was used for the determination of esterase, GST and ChE activities. The microsome precipitation was dissolved in 1 mL homogenization buffer and used for measuring cytochrome P450 monooxygenases activity. The protein concentration was determined by a BCA protein kit (Sangon Biotech). Three replicates for each of the four treatments (MT, MC, AT, and AC) were conducted and processed independently.

P450 PNOD activity was performed according to previously published procedures with slight modifications^56^. One hundred μL of microsome protein was added to 100 μL of 2 mM p-nitroanisole, and the mixture was incubated at 30 °C for 3 min. Then, the reaction was initiated by the addition of 20 μL of 10 mM NADPH. After 30 min at 30 °C, 100 μL HCL (1 mol/L) was added to stop the reaction. For blank control, proteins were added after HCL. Next, 500 μL chloroform was added to the mixture to extract the reaction product. Three hundred μL chloroform layer was transferred to a new tube and 300 μL NaOH (0.5 mol/L) was added. After shaking and centrifuging, 200 μL aqueous phase was read in a spectrophotometer at 405 nm. A p-nitrophenol standard curve was used to quantify product. The activity was expressed as nmole p-nitrophenol per minute per milligram protein.

Esterase activity towards α-naphthyl acetate (α-NA) was conducted as preciously described^57^. One hundred μL total proteins was diluted by adding 2.9 mL sodium phosphate buffer (0.1 M pH 7.8), and then mixed gently with 1 mL substrate solution (0.1 M pH 7.8 phosphate buffer, 0.3 mM α-NA). After incubation at 37 °C for 15 min, 1 mL colour reagent (2 mL 1% fast blue B salt mixed with 5 mL 5% SDS) was added to stop the reaction. Ten min after the coloration, the absorbance was measured at 600 nm. For blank control, proteins were added after colour reagent. A naphthol standard curve was used to quantify product. The esterase activity was expressed as nmole naphthol per minute per milligram protein.

ChE activity was detected by using a ChE detection kit (Nanjing Jiancheng Bioengineering). After incubation at 37 °C for 20 min, the residual acetylcholine was detected by reaction with hydroxylamine and then mixed with Fe^2+^ in acid solution for coloration. The absorbance was measured at 520 nm and the activity was expressed as nmole acetylcholine decreased per minute per milligram protein.

GST activity towards 3, 4-dichloronitrobenzene (DCNB) was assayed using GSH-ST detection kit (Nanjing Jiancheng Bioengineering). After incubation at 37 °C for 10 min, the residual L-glutathione (GSH) was detected by reaction with the general thiol reagent (5-5’-dithiobis [acid], DTNB). The absorbance of the reaction product 5-thionitrobenzoic acid (TNB) was measured at 412 nm. GST activity was normalized per milligram of protein. One unit of GST activity was defined as the formation of 1 nmol of CDNB-GSH per minute of incubation.

### Data accessibility

Data sets are available at the NCBI Gene Expression Omnibus (GEO) with the accession number: GSE57074. The following link has been created to allow review of record GSE57074 while it remains in private status: http://www.ncbi.nlm.nih.gov/geo/query/acc.cgi?token=atcjycwgzjmnvod&acc=GSE57074

## Additional Information

**How to cite this article**: Xu, H.-X. *et al.* Transcriptional responses of invasive and indigenous whiteflies to different host plants reveal their disparate capacity of adaptation. *Sci. Rep.* **5**, 10774; doi: 10.1038/srep10774 (2015).

## Supplementary Material

Supplementary Information

Supplementary Table S1

Supplementary Table S2

Supplementary Table S3

Supplementary Table S4

Supplementary Table S5

Supplementary Table S6

Supplementary Table S7

## Figures and Tables

**Table 1:** Statistics of DGE sequencing.

**Category**	**Parameter**	**Sequencing results**			
		**MC**^1^	**MT**^2^	**AC**^3^	**AT**^4^
Raw tag	Total no. of tags	5951527	6091042	6114520	5879545
	No. of distinct tags	342727	318139	247567	266527
Clean tag	Total number	5734967	5888090	5976544	5730194
	Distinct Tag number	143198	133826	111624	119204
All tags mapping to gene	No. of distinct tags	43787	47908	35183	23368
	Distinct Tag % of clean tag	30.58%	35.80%	31.52%	19.60%
All tag-mapped genes	No. of genes	22193	22620	18098	13828
	% of reference genes	38.44%	39.17%	34.45%	26.32%
Unambiguous tag-mapped genes	No. of genes	22113	22547	18006	13734
	% of reference genes	38.30%	39.05%	34.27%	26.14%

^1^The MEAM1 whitefly transferred from cotton to cotton.

^2^The MEAM1 whitefly transferred from cotton to tobacco.

^3^The Asia II 3 whitefly transferred from cotton to cotton.

^4^The Asia II 3 whitefly transferred from cotton to tobacco.

**Table 2:** Pathways enriched with DEGs in MT vs. MC and AT vs. AC comparison groups.

**Pathway ID**	**Pathway description**	**Dif**^1^	**Ref**^2^	**P-value**
**MT vs. MC**				
ko04142	Lysosome	20	948	3.10E-06
ko04612	Antigen processing and presentation	12	389	8.18E-06
ko03013	RNA transport	15	927	0.0009172
ko03050	Proteasome	5	187	0.0069723
ko00330	Arginine and proline metabolism	5	229	0.0157006
ko00340	Histidine metabolism	3	98	0.0246248
ko00620	Pyruvate metabolism	5	281	0.0339742
ko00561	Glycerolipid metabolism	5	312	0.0493495
**AT vs. AC**				
ko00190	Oxidative phosphorylation	38	399	2.48E-21
ko03010	Ribosome	28	478	7.26E-11
ko01100	Metabolic pathways	131	6447	1.15E-07
ko03050	Proteasome	14	187	2.28E-07
ko01120	Microbial metabolism in diverse environments	38	1604	0.00042
ko03060	Protein export	6	118	0.004905
ko00980	Metabolism of xenobiotics by cytochrome P450	15	531	0.005158
ko00062	Fatty acid elongation in mitochondria	4	54	0.005656
ko00982	Drug metabolism - cytochrome P450	15	537	0.005701
ko00240	Pyrimidine metabolism	17	647	0.006134
ko00280	Valine, leucine and isoleucine degradation	9	274	0.011196
ko00020	Citrate cycle (TCA cycle)	8	229	0.011624
ko04141	Protein processing in endoplasmic reticulum	22	990	0.013199
ko04612	Antigen processing and presentation	11	389	0.015233
ko00010	Glycolysis / Gluconeogenesis	8	241	0.015387
ko00480	Glutathione metabolism	9	305	0.020979
ko05130	Pathogenic Escherichia coli infection	9	305	0.020979
ko03020	RNA polymerase	8	265	0.025434
ko00640	Propanoate metabolism	6	185	0.036956
ko00030	Pentose phosphate pathway	6	187	0.038618
ko00350	Tyrosine metabolism	8	292	0.041385
ko00450	Selenocompound metabolism	5	143	0.041797
ko04621	NOD-like receptor signaling pathway	5	145	0.043921
ko05100	Bacterial invasion of epithelial cells	9	357	0.049153

^1^The number of DEGs in a pathway.

^2^The total number of genes in a pathway.
