# Supplementary material for: Transcriptional responses of invasive and indigenous whiteflies to different host plants reveal their disparate capacity of adaptation
Source: Sci Rep. 2015 Jun 4;5:10774. doi: 10.1038/srep10774 (PMC4455138; doi:10.1038/srep10774)
Supplement: Supplementary Information [file srep10774-s1.pdf]

Transcriptional responses of invasive and indigenous whiteflies to different host plants reveal their disparate capacity of adaptation

Hong-Xing Xu, Yue Hong, Min-Zhu Zhang, Yong-Liang Wang, Shu-Sheng Liu, Xiao-Wei Wang

Supplementary Dataset 1: Differently expressed genes between cotton-feeding and tobacco-feeding MEAM1.

Supplementary Dataset 2: Differently expressed genes between cotton-feeding and tobacco-feeding Asia II 3.

Supplementary Dataset 3: Comparison of DGE data and qRT-PCR results for 50 genes. Twenty-five DEGs from each comparison group (MT vs. MC and AT vs. AC) were selected for qRT-PCR analyses to validate the DGE data.

Supplementary Dataset 4: Carbohydrate and energy metabolism pathways enriched with DEGs in AT vs. AC.

Supplementary Dataset 5: DEGs in the ribosome pathway in AT vs. AC and MT vs. MC.

Supplementary Dataset 6: DEGs in proteasome, lysosome and protein digestion and absorption pathways in AT vs. AC and MT vs. MC.

Supplementary Dataset 7: Differently expressed detoxification genes in AT vs. AC and MT vs. MC.
